# Supplementary material for: Menoci: lightweight extensible web portal enhancing data management for biomedical research projects
Source: BMC Bioinformatics. 2020 Dec 17;21:582. doi: 10.1186/s12859-020-03928-1 (PMC7745495; doi:10.1186/s12859-020-03928-1)
Supplement: Supplementary file 1 — Additional file 1: menoci Graphical Guide. The additional file contains multiple screenshots taken from menoci instances operated by the authors' group. The use case of collecting and documenting antibody and advanced light microscopy data is depicted to give an impression of how menoci puts data management and experiment workflow support into practise. [file 12859_2020_3928_MOESM1_ESM.pdf]

Supplement 1: menoci Graphical Guide

The following pages contain a series of screenshots taken from menoci instances used to support data management for the Collaborative Research Centre 1002.
Infomation visible in some pictures may have been altered or blurred because it has not been cleared for publication by the original owners or creators.

The use case depicted is designed to roughly illustrate how menoci supports data management in an advanced light microscopy setting, i.e. how antibodies used for microscopy staining can be documented and referred to in the system. Finally, result data from the experiment is uploaded and the relation to a published scientific article is documented.

CC-BY-SA 4.0
menoci.io
Suhr M, Lehmann C, Bauer CR, Bender T, Knopp C, Freckmann LK, Öst Hansen B, Henke C, Aschenbrandt G, Kühlborn LK, Rheinländer S, Weber L, Marzec B, Hellkamp M, Wieder P, Sax U, Kusch H, Nussbeck SY

1. Overview of menoci instance start page

The start page of the CRC1002 menoci instance allows access to four menoci modules for non-authorized users: Published Data Registry, Antibody Catalogue, Lab Notebook Registry and Cell Model Catalogue. Following successful login, more modules are available. The startpage further contains a project description as well as contact information for the project speaker, participating instutions and funding agency.

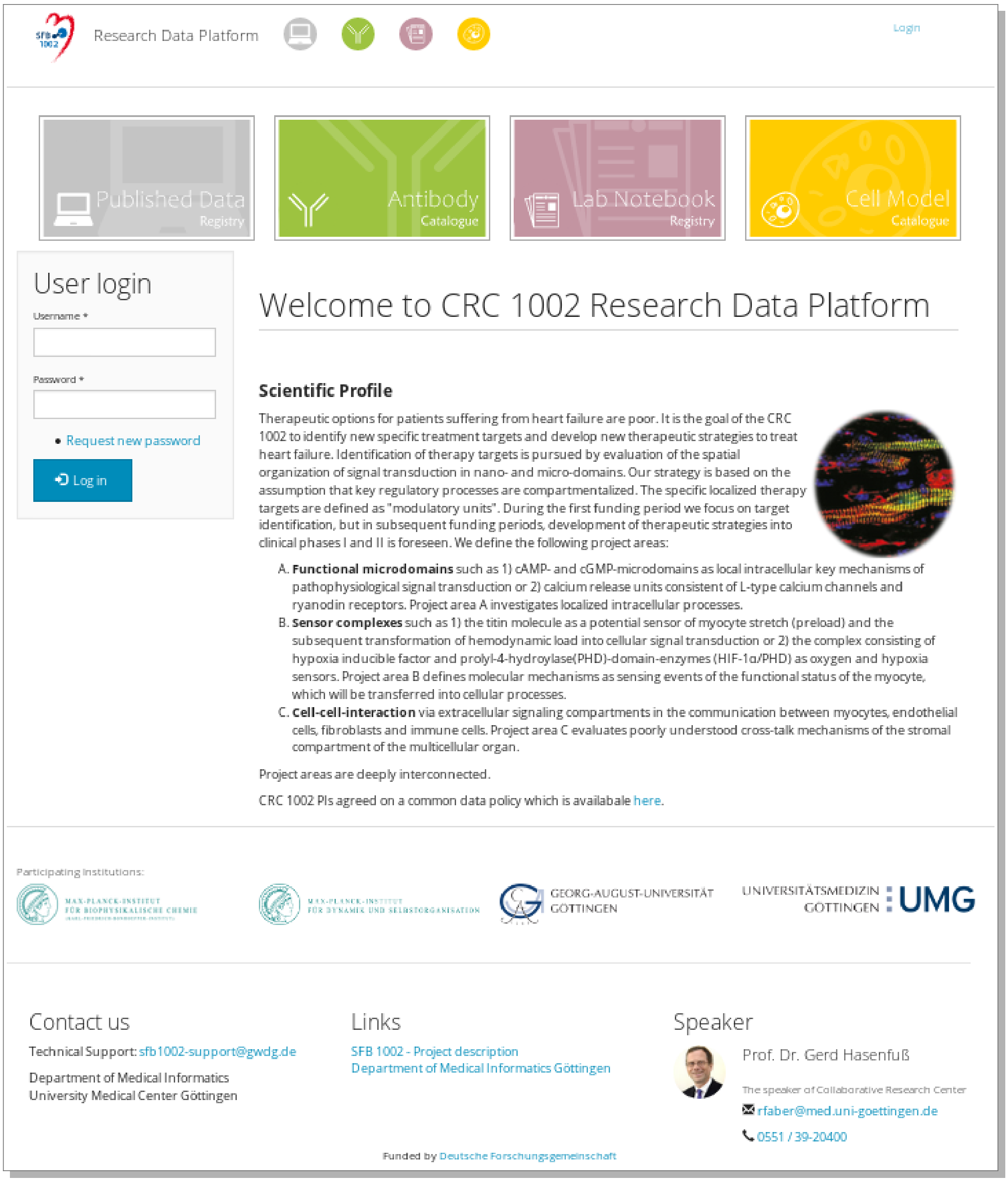

CC-BY-SA 4.0

Suhr M, Lehmann C, Bauer CR, Bender T, Knopp C, Freckmann LK, Öst Hansen B, Henke C, Aschenbrandt G, Kühlborn LK, Rheinländer S, Weber L, Marzec B, Hellkamp M, Wieder P, Sax U, Kusch H, Nussbeck SY

2. Documentation of antibodies

When accessing the Antibody Catalogue module (here as an already logged in user), antibodies registered with the system are displayed split into categories of primary and secondary antibodies. A searchbar allows for quick access to specific information. All data available for the current user based on access privileges may be exported in spreadsheet format. If authorized accordingly, new antibody infomation may be registered by the user.

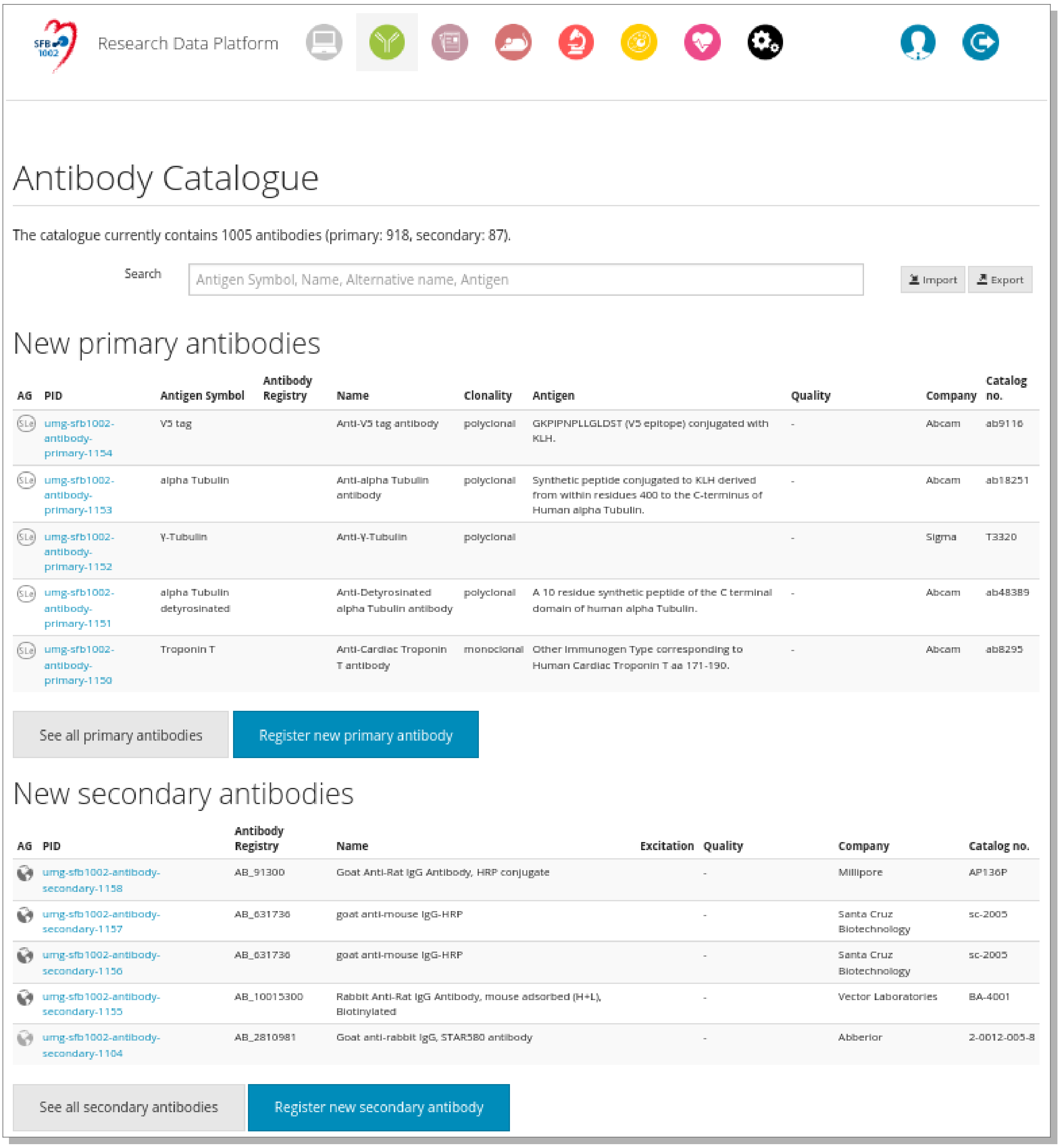

CC-BY-SA 4.0

Suhr M, Lehmann C, Bauer CR, Bender T, Knopp C, Freckmann LK, Öst Hansen B, Henke C, Aschenbrandt G, Kühlborn LK, Rheinländer S, Weber L, Marzec B, Hellkamp M, Wieder P, Sax U, Kusch H, Nussbeck SY

When inserting new antibody data, multiple descriptive metadata are collected from the user, including manufacturer, catalogue nr., Lot number, and on-site storage information.

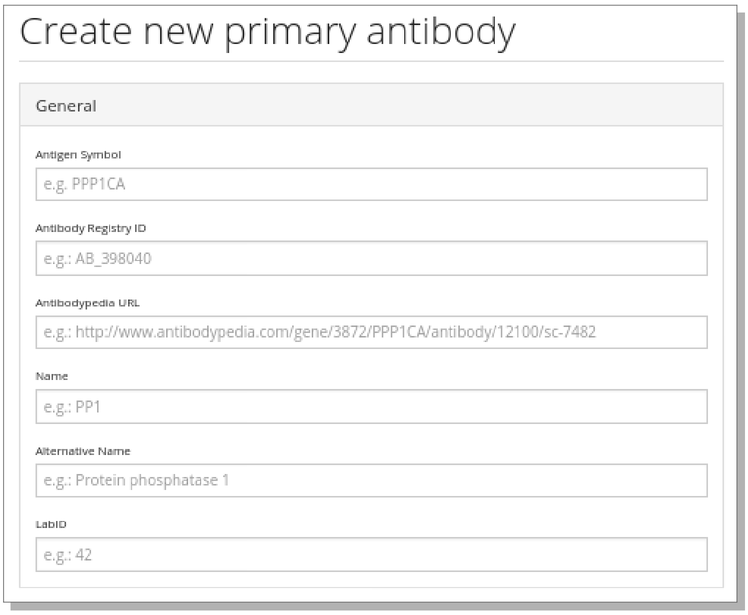

Finally, the user is asked to specificy which of the project‘s research groups is in possession of the described antibody and which „Sharing level“ shall be assigned. Four different „Sharing levels“ allow distinction between publically visible data, project-wide, research group-internal, or private data. These same levels provide users of the menoci system with a simple recurring classification of data visibility.

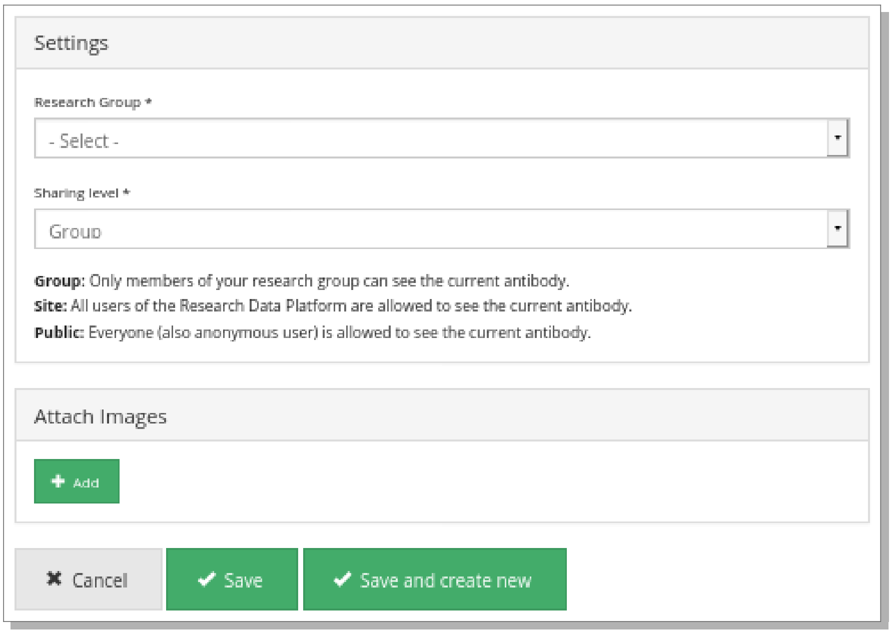

Images may be uploaded and annotated with comments, for example when further detailing antibody performance in Western Blot.

CC-BY-SA 4.0

Suhr M, Lehmann C, Bauer CR, Bender T, Knopp C, Freckmann LK, Öst Hansen B, Henke C, Aschenbrandt G, Kühlborn LK, Rheinländer S, Weber L, Marzec B, Hellkamp M, Wieder P, Sax U, Kusch H, Nussbeck SY

A newly created antibody item will be assigned an internal identifier. When cooperating with a Handle.net compatible Persistent Identifier (PID) provider (i.e. the EPIC consortium), internal IDs may be registered permanently and used for public reference. The PID is display at the top of antibody item display page.

The inserted information may be retrieved as a JSON or PDF document.

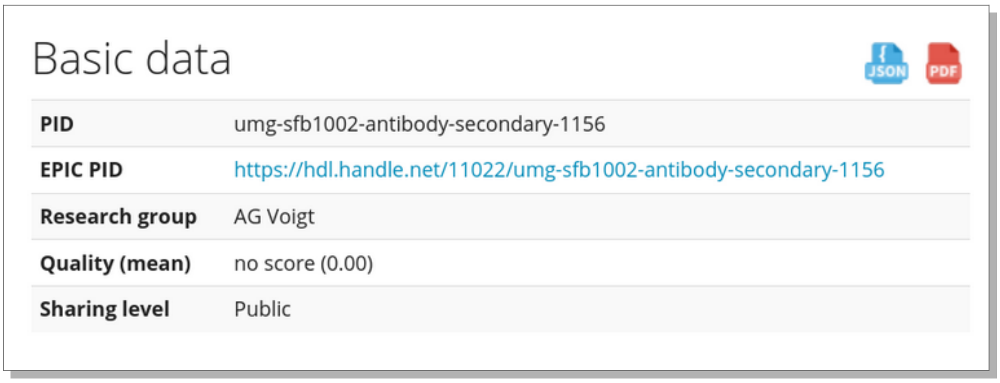

Once an antibody has been registered and used in multiple experiments, users have the possibility of adding performance quality rankings. A simple 0-5 star ranking system allows other users to quickly assess how well a certain antibody has performed in their colleagues‘ experiments.

Furthermore, publications registered with the menoci instance that refer to the selected antibody are displayed on the right.

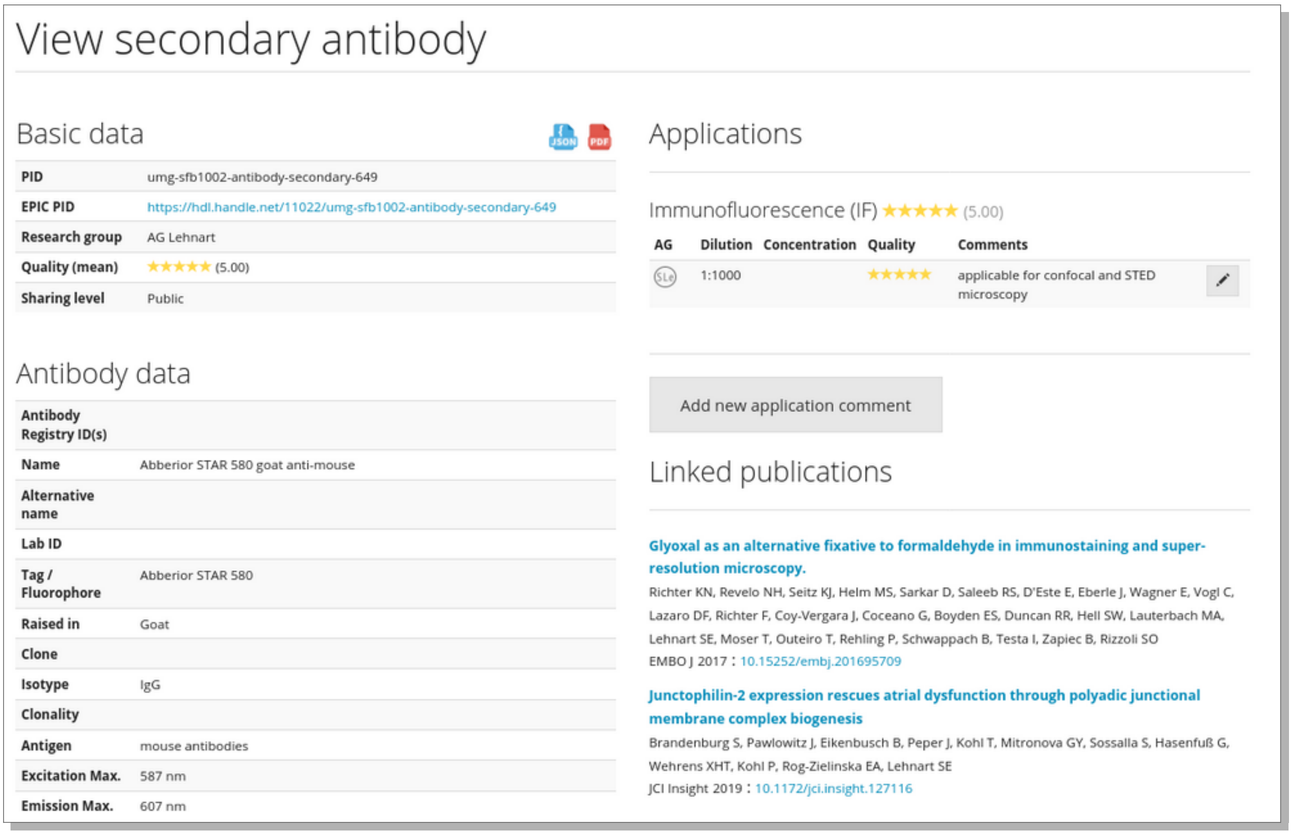

CC-BY-SA 4.0

Suhr M, Lehmann C, Bauer CR, Bender T, Knopp C, Freckmann LK, Öst Hansen B, Henke C, Aschenbrandt G, Kühlborn LK, Rheinländer S, Weber L, Marzec B, Hellkamp M, Wieder P, Sax U, Kusch H, Nussbeck SY

3. Documentation of microscopy experiment

The CRC1002 instance of menoci contains the „Advanced Light Microscopy and Nanoscopy“ (ALMN) module that provides workflow support for the local imaging service unit.

Documentation of microscopy experiments is structured accordingly into „requests“ by users, „consultation“ documentation by service unit personnel, and data upload functionality for the user.

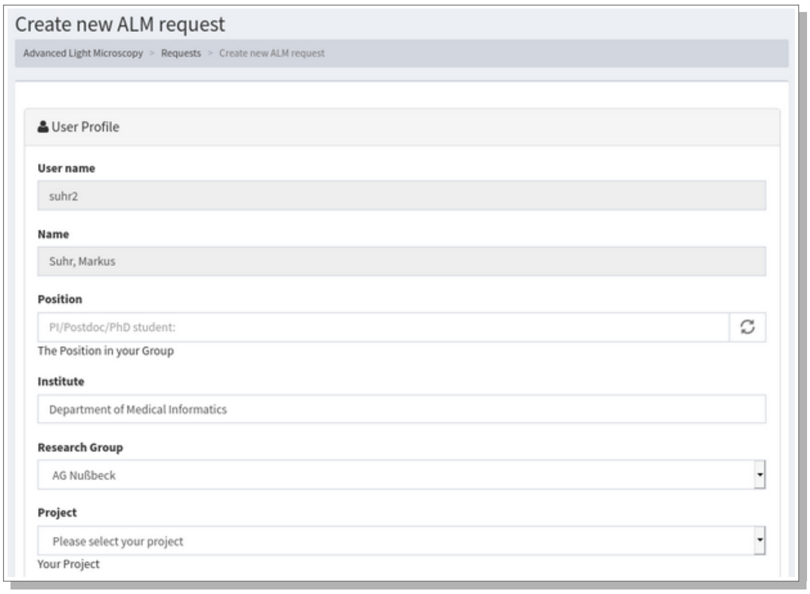

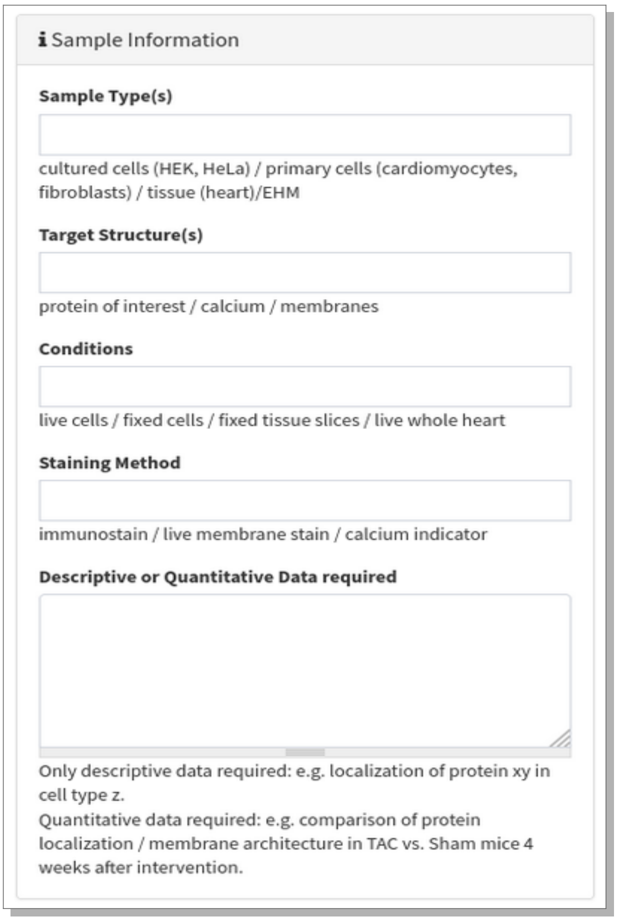

Informaton entered when creating a new request spans basic user information as well as a description of the experiment goals and parameters.

CC-BY-SA 4.0

Suhr M, Lehmann C, Bauer CR, Bender T, Knopp C, Freckmann LK, Öst Hansen B, Henke C, Aschenbrandt G, Kühlborn LK, Rheinländer S, Weber L, Marzec B, Hellkamp M, Wieder P, Sax U, Kusch H, Nussbeck SY

During consultation phase, more in-depth information about the planned imaging procedure are captured. Antibody data from the Antibody Catalogue is referenced to describe and document the prepared stainings.

Dual Immunofluorescence Stainings

+Add new

Short name for label: SUR1 PKM 86

1	primary antibody	dilution	secondary antibody	dilution
	**Antigen Symbol:** ABCC8 **Name:** SUR1<br>**Raised in:** mouse **Catalog No.:** 73-267<br>umg-sfb1002-antibody-primary-53	1:200	**Antigen Symbol: Name:** Abberior STAR 635P goat anti-mouse<br>**Raised in:** Goat **Catalog No.:** 2-0002-007-5<br>umg-sfb1002-antibody-secondary-647	1:1000
	**Antigen Symbol:** PKM2 **Name:** Pyruvatekinase<br>**Catalog No.:** 15821-1-AP<br>umg-sfb1002-antibody-primary-678	1:1000	**Antigen Symbol: Name:** Abberior STAR 580 goat anti-mouse<br>**Raised in:** Goat **Catalog No.:** 2-0002-005-1<br>umg-sfb1002-antibody-secondary-649	1:1000
	-	-	-	-

Short name for label: SUR1 ENO1 84

2	primary antibody	dilution	secondary antibody	dilution
	**Antigen Symbol:** ABCC8 **Name:** SUR1<br>**Raised in:** mouse **Catalog No.:** 73-267<br>umg-sfb1002-antibody-primary-53	1:200	**Antigen Symbol: Name:** Abberior STAR 635P goat anti-mouse<br>**Raised in:** Goat **Catalog No.:** 2-0002-007-5<br>umg-sfb1002-antibody-secondary-647	1:1000
	**Antigen Symbol:** Eno1 **Name:** Alpha Enolase<br>**Raised in:** rabbit **Catalog No.:** LS-C482249<br>umg-sfb1002-antibody-primary-681	1:500	**Antigen Symbol: Name:** Abberior STAR 580 goat anti-rabbit<br>**Raised in:** goat **Catalog No.:** 2-0012-005-8<br>umg-sfb1002-antibody-secondary-650	1:1000
	-	-	-	-

Furthermore, samples used in the imaging workflow will be documentated.

Samples

+Add new

1		
Serial #		00017
Species	mouse	m
ID		
Type	atrial myocytes	AM
Genotype	Wild Type	WT
Treatment		
Staining type	IF	IF

2		
Serial #		00018
Species	mouse	m

CC-BY-SA 4.0

Suhr M, Lehmann C, Bauer CR, Bender T, Knopp C, Freckmann LK, Öst Hansen B, Henke C, Aschenbrandt G, Kühlborn LK, Rheinländer S, Weber L, Marzec B, Hellkamp M, Wieder P, Sax U, Kusch H, Nussbeck SY

The specific setup at the local Göttingen ALMN facility enables users to print labels for the actual microscopy coverslips. Labels contain a barcode that resolves to a Handle.net registered PID with is unique for each printed label and refers back to the ALMN project.

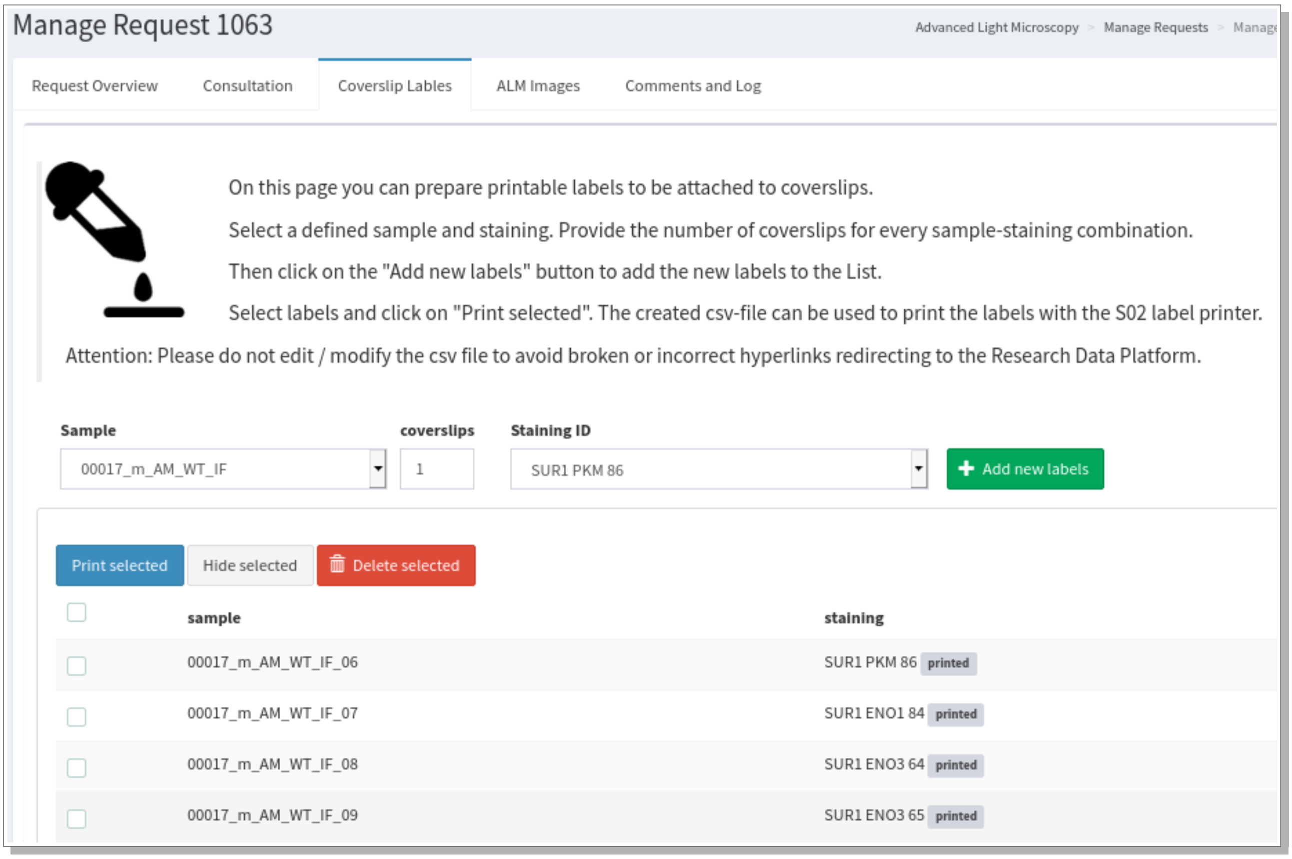

CC-BY-SA 4.0

Suhr M, Lehmann C, Bauer CR, Bender T, Knopp C, Freckmann LK, Öst Hansen B, Henke C, Aschenbrandt G, Kühlborn LK, Rheinländer S, Weber L, Marzec B, Hellkamp M, Wieder P, Sax U, Kusch H, Nussbeck SY

4. Upload of result data

As soon as the microscopic imaging has been performed, users may upload datasets for result documentation and original data preservation. Data uploaded is stored directly in an instance of the CDSTAR storage service that is managed by the Göttingen Campus scientific data centre in case of the CRC1002. Users do not have to put in further effort to store and archive their original data in this setup.

Data uploaded can be accessed by the owner, downloaded, deleted. General access rights may be set according to the common menoci „Sharing levels“ (public, site, group, private).

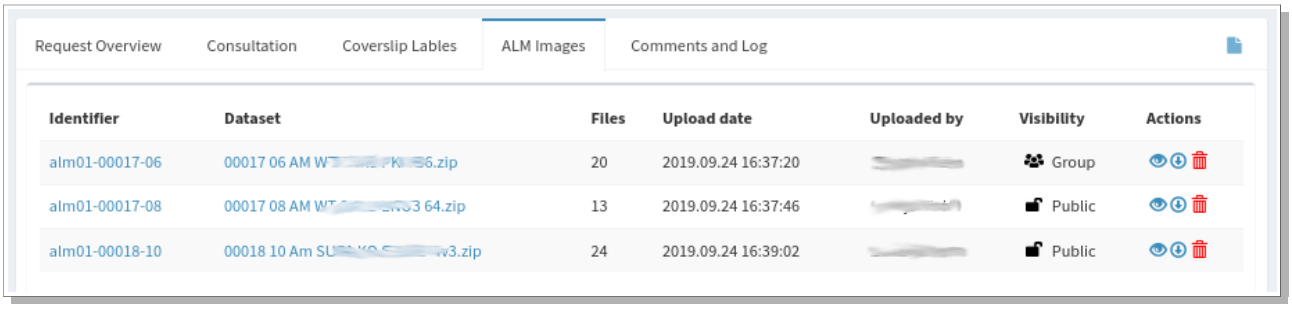

Uploaded data is assigned a Handle.net PID and can be further shared with researchers from specific other research groups within the menoci instance. Previews of uploaded XML and image files are provided.

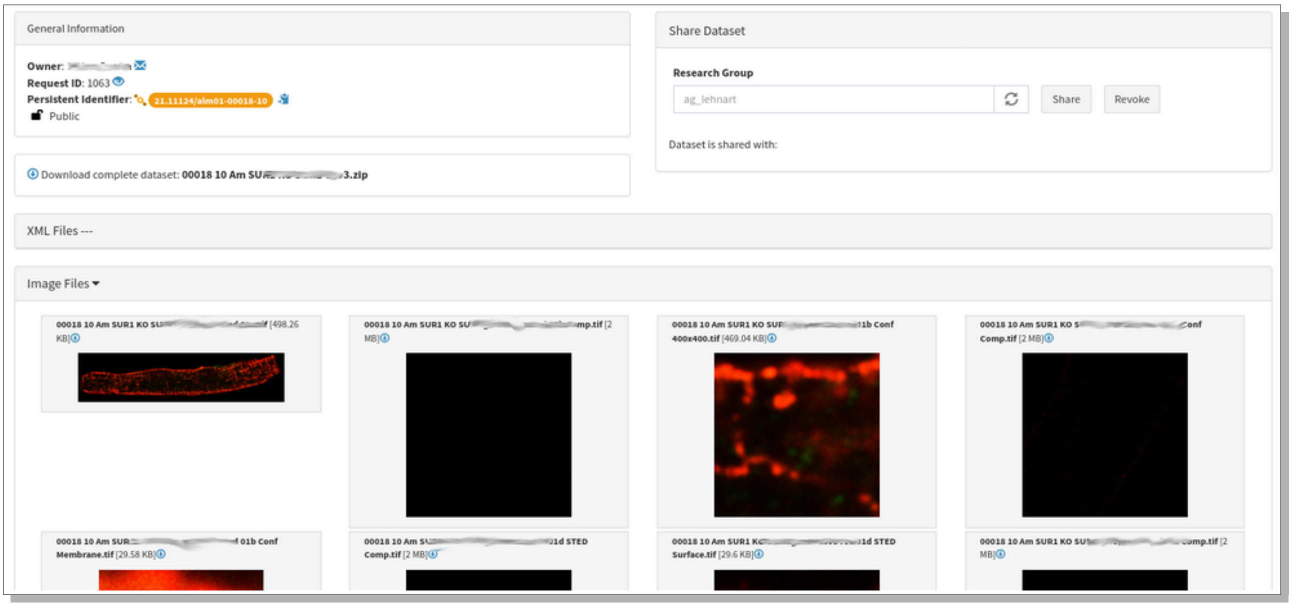

CC-BY-SA 4.0

Using the registered Handle.net PID, metadata about the ALMN project is publicly accessible. If uploaded data has been designated „public“ by the user, files may be downloaded by non-authorized users.

For example, visit: http://hdl.handle.net/21.11124/alm01-00017-06

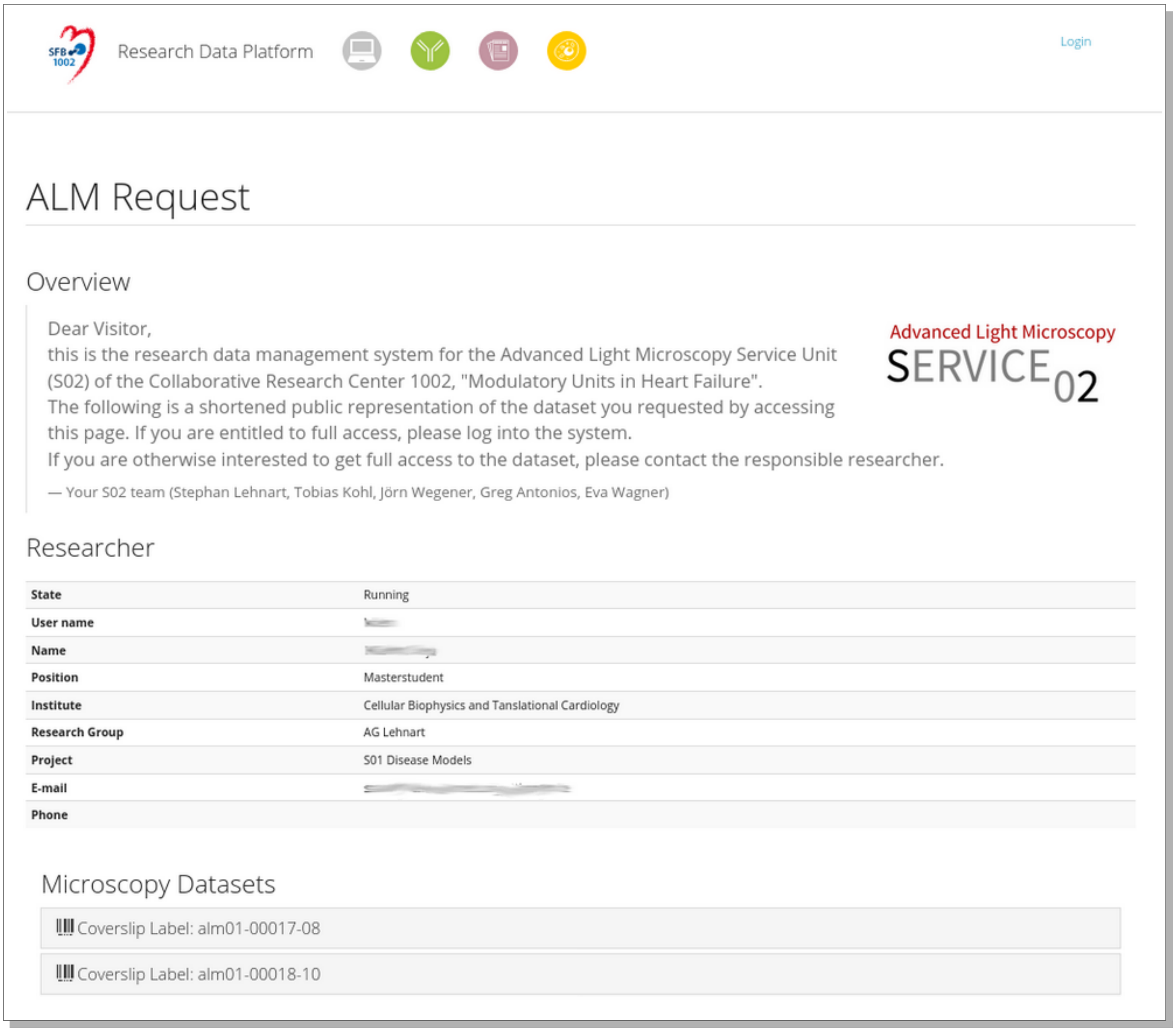

CC-BY-SA 4.0

Suhr M, Lehmann C, Bauer CR, Bender T, Knopp C, Freckmann LK, Öst Hansen B, Henke C, Aschenbrandt G, Kühlborn LK, Rheinländer S, Weber L, Marzec B, Hellkamp M, Wieder P, Sax U, Kusch H, Nussbeck SY

5. Registration of published article

As soon as research results have been published with a scientific journal or uploaded to a preprint server, articles can be added to the menoci Published Data Registry for the project.

Initially, an overview of all registered articles is displayed with relevant information at a glance, including DOI, PubMeD IDs.

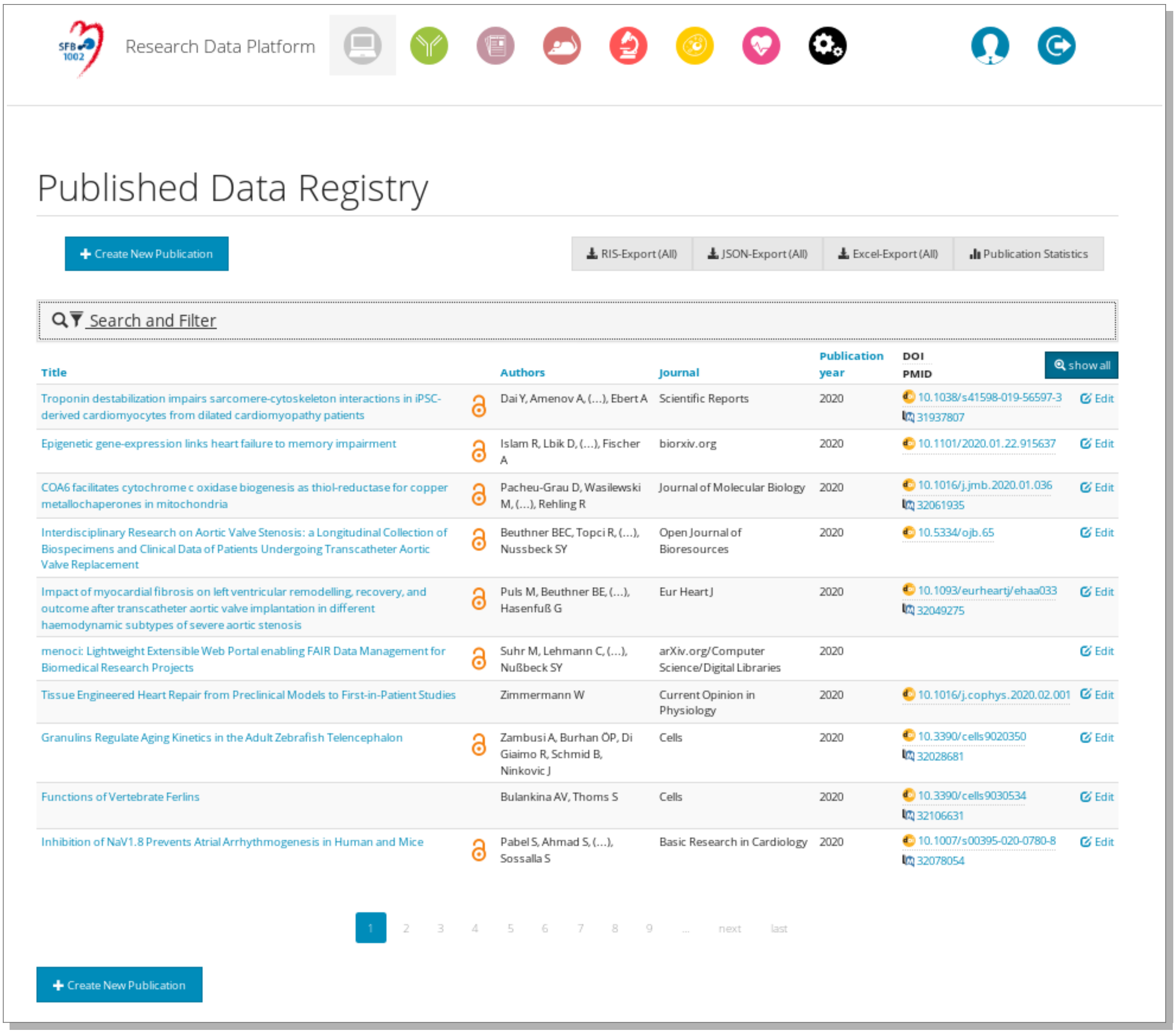

Title	Authors	Journal	Publication year	DOI PMID	
Troponin destabilization impairs sarcomere-cytoskeleton interactions in iPSC-derived cardiomyocytes from dilated cardiomyopathy patients	Dai Y, Amenov A, (...), Ebert A	Scientific Reports	2020	10.1038/s41598-019-56597-3<br>31937807	Edit
Epigenetic gene-expression links heart failure to memory impairment	Islam R, Lbik D, (...), Fischer A	biorxiv.org	2020	10.1101/2020.01.22.915637	Edit
COA6 facilitates cytochrome c oxidase biogenesis as thiol-reductase for copper metallochaperones in mitochondria	Pacheu-Grau D, Wasilewski M, (...), Rehling R	Journal of Molecular Biology	2020	10.1016/j.jmb.2020.01.036<br>32061935	Edit
Interdisciplinary Research on Aortic Valve Stenosis: a Longitudinal Collection of Biospecimens and Clinical Data of Patients Undergoing Transcatheter Aortic Valve Replacement	Beuthner BEC, Topci R, (...), Nussbeck SY	Open Journal of Bioresources	2020	10.5334/ojb.65	Edit
Impact of myocardial fibrosis on left ventricular remodelling, recovery, and outcome after transcatheter aortic valve implantation in different haemodynamic subtypes of severe aortic stenosis	Puls M, Beuthner BE, (...), Hasenfuß G	Eur Heart J	2020	10.1093/eurheartj/ehaa033<br>32049275	Edit
menoci: Lightweight Extensible Web Portal enabling FAIR Data Management for Biomedical Research Projects	Suhr M, Lehmann C, (...), Nußbeck SY	arXiv.org/Computer Science/Digital Libraries	2020		Edit
Tissue Engineered Heart Repair from Preclinical Models to First-in-Patient Studies	Zimmermann W	Current Opinion in Physiology	2020	10.1016/j.cophys.2020.02.001	Edit
Granulins Regulate Aging Kinetics in the Adult Zebrafish Telencephalon	Zambusi A, Burhan ÖP, Di Giaimo R, Schmid B, Ninkovic J	Cells	2020	10.3390/cells9020350<br>32028681	Edit
Functions of Vertebrate Ferlins	Bulankina AV, Thoms S	Cells	2020	10.3390/cells9030534<br>32106631	Edit
Inhibition of NaV1.8 Prevents Atrial Arrhythmogenesis in Human and Mice	Pabel S, Ahmad S, (...), Sossalla S	Basic Research in Cardiology	2020	10.1007/s00395-020-0780-8<br>32078054	Edit

1 2 3 4 5 6 7 8 9 ... next last

Create New Publication

CC-BY-SA 4.0

Suhr M, Lehmann C, Bauer CR, Bender T, Knopp C, Freckmann LK, Öst Hansen B, Henke C, Aschenbrandt G, Kühlborn LK, Rheinländer S, Weber L, Marzec B, Hellkamp M, Wieder P, Sax U, Kusch H, Nussbeck SY

Furthermore, basic live statics of the project publication record are publicly available.

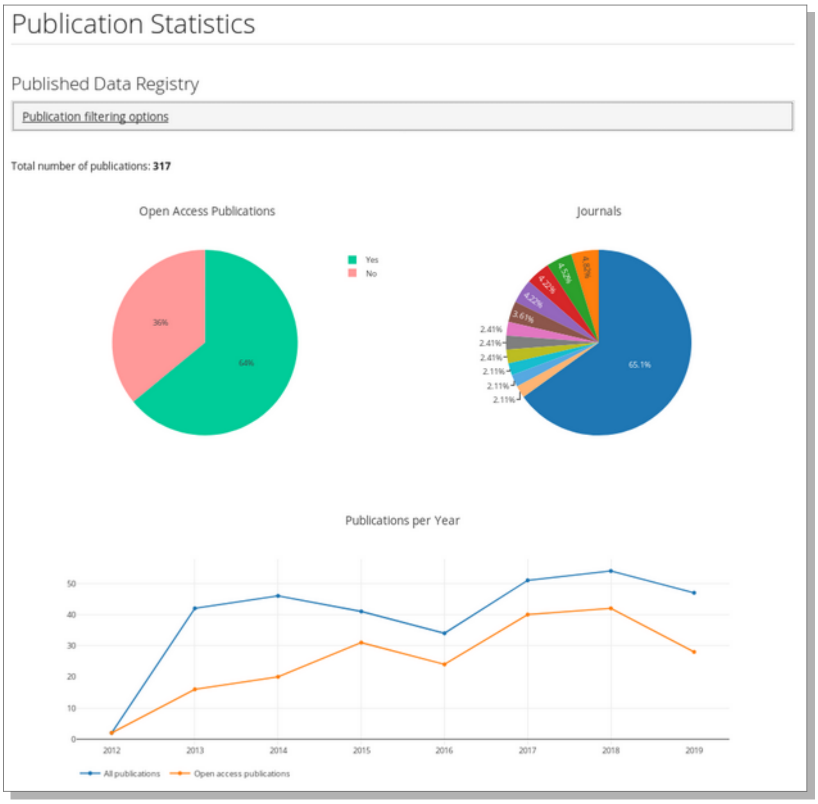

Creating a new entry to the Published Data Registry can be streamlined if the article is already published and assigned a DOI or PubMed ID.

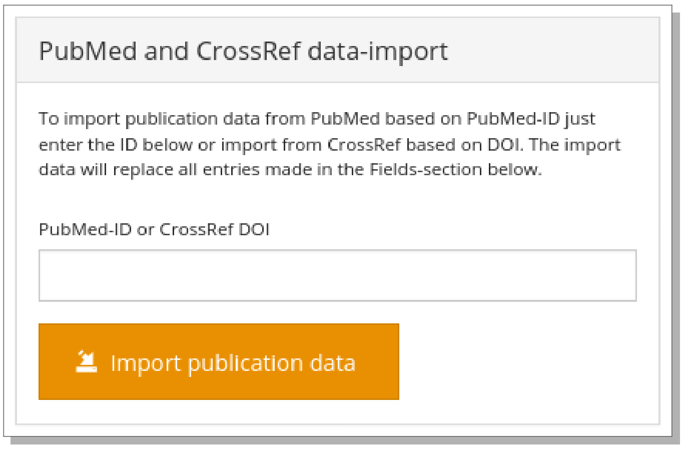

Otherwise, relevant metadata must be inserted using a basic web form.

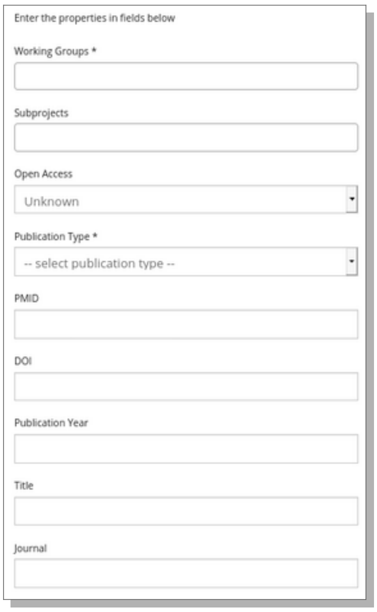

CC-BY-SA 4.0

Suhr M, Lehmann C, Bauer CR, Bender T, Knopp C, Freckmann LK, Öst Hansen B, Henke C, Aschenbrandt G, Kühlborn LK, Rheinländer S, Weber L, Marzec B, Hellkamp M, Wieder P, Sax U, Kusch H, Nussbeck SY

Once completed, inserted article information is publicly visible at the website. Additional information may be inserted according to instance configuration under the “External Resources” section. This may be used to refer to supplemental material, biomaterial accession numbers at public data repositories, or any other kind of URL-identifiable resource.

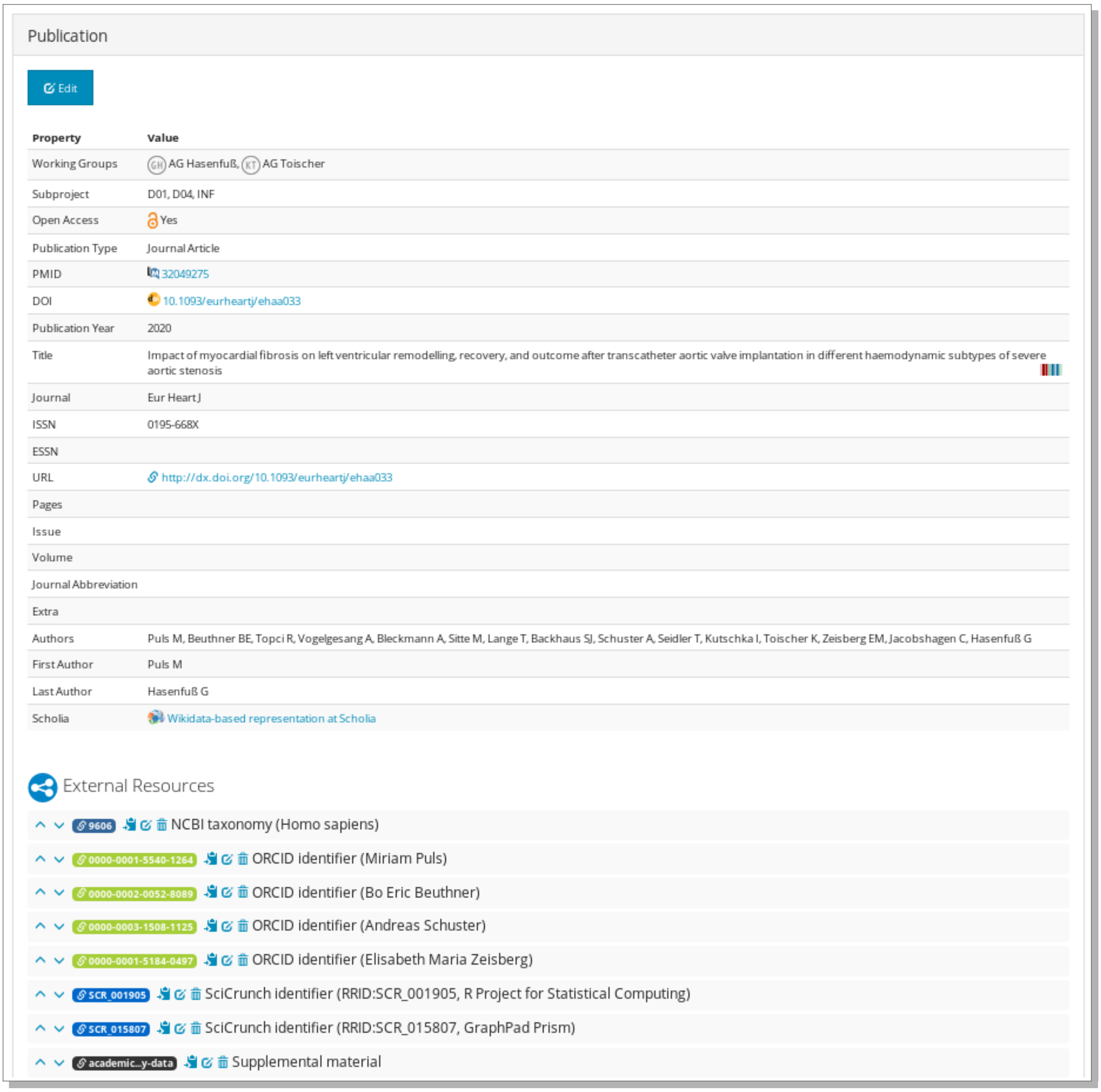

Publication

Edit

Property	Value
Working Groups	AG Hasenfuß, AG Toischer
Subproject	D01, D04, INF
Open Access	Yes
Publication Type	Journal Article
PMID	32049275
DOI	10.1093/eurheartj/ehaa033
Publication Year	2020
Title	Impact of myocardial fibrosis on left ventricular remodelling, recovery, and outcome after transcatheter aortic valve implantation in different haemodynamic subtypes of severe aortic stenosis
Journal	Eur Heart J
ISSN	0195-668X
ESSN	
URL	http://dx.doi.org/10.1093/eurheartj/ehaa033
Pages	
Issue	
Volume	
Journal Abbreviation	
Extra	
Authors	Puls M, Beuthner BE, Topci R, Vogelgesang A, Bleckmann A, Sitte M, Lange T, Backhaus SJ, Schuster A, Seidler T, Kutschka I, Toischer K, Zeisberg EM, Jacobshagen C, Hasenfuß G
First Author	Puls M
Last Author	Hasenfuß G
Scholia	Wikidata-based representation at Scholia

External Resources

- 9606 NCBI taxonomy (Homo sapiens)
- 0000-0001-5540-1264 ORCID identifier (Miriam Puls)
- 0000-0002-0052-8089 ORCID identifier (Bo Eric Beuthner)
- 0000-0003-1508-1125 ORCID identifier (Andreas Schuster)
- 0000-0001-5184-0497 ORCID identifier (Elisabeth Maria Zeisberg)
- SCR_001905 SciCrunch identifier (RRID:SCR_001905, R Project for Statistical Computing)
- SCR_015807 SciCrunch identifier (RRID:SCR_015807, GraphPad Prism)
- academic...y-data Supplemental material

6. Documentation of original data related to publications

Published article information can finally be enriched by providing references to original experimental resources like Antibodies as well as microscopy imaging result data.

Authorized users are presented with searchbars that allow item search and selection:

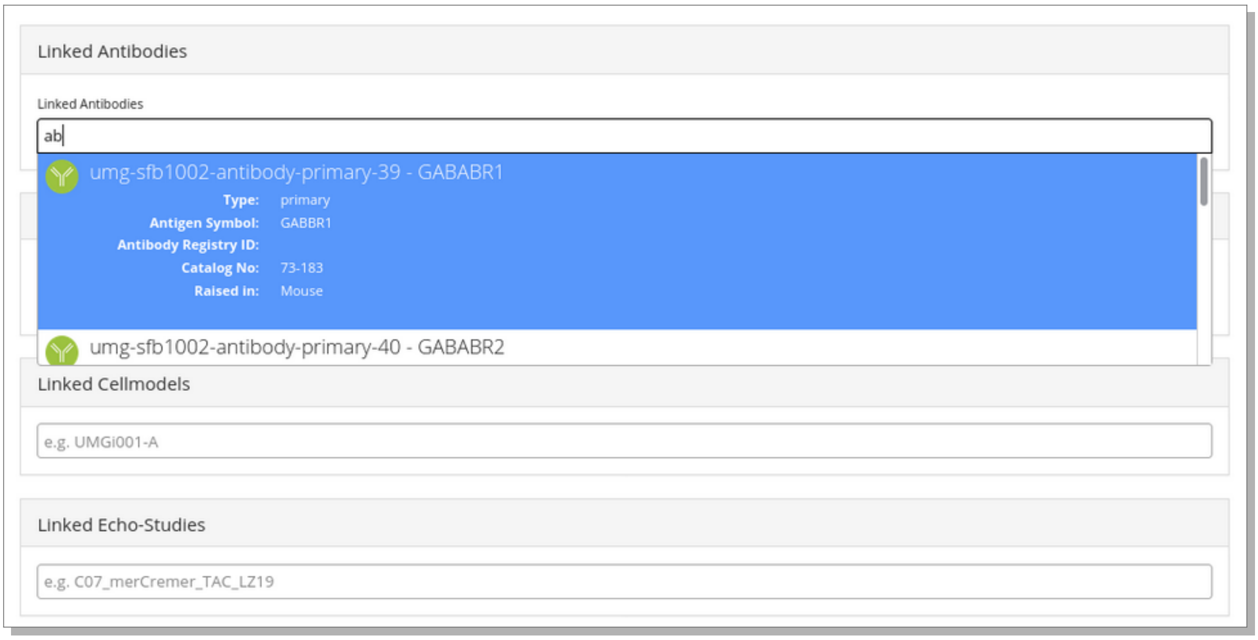

Such linked resources will in turn be publicly displayed at the article page:

Linked Antibodies

PID	AG	Type	Antigen symbol	Antibody Registry ID	Name
umg-sfb1002-antibody-primary-193	SLe	primary	RYR2	AB_2183054	Ryanodine Receptor 2
umg-sfb1002-antibody-primary-922	SLe	primary		AB_1163685	IgG1 Isotype Control from murine myeloma
umg-sfb1002-antibody-primary-923	SLe	primary	PPP1CC	AB_2299993	PP1c
umg-sfb1002-antibody-primary-924	SLe	primary	PPP1R3A		PPP1R3A
umg-sfb1002-antibody-primary-925	SLe	primary		AB_737167	normal goat IgG
umg-sfb1002-antibody-primary-926	SLe	primary	PPP1R3A	AB_11014462	PPP1R3A
umg-sfb1002-antibody-primary-927	SLe	primary	PPP2CA	AB_397909	Purified Mouse Anti-PP2A Catalytic α
umg-sfb1002-antibody-primary-928	SLe	primary	ATP2A2	AB_325502	SERCA2a
umg-sfb1002-antibody-primary-929	SLe	primary	CASQ2	AB_2071461	Calsequestrin
umg-sfb1002-antibody-primary-930	SLe	primary	PLN	AB_2252716	Phospholamban
umg-sfb1002-antibody-primary-931	SLe	primary	PLN	AB_2617049	Phospholamban (PLN, PLB) mAb (clone A1)

CC-BY-SA 4.0

Suhr M, Lehmann C, Bauer CR, Bender T, Knopp C, Freckmann LK, Öst Hansen B, Henke C, Aschenbrandt G, Kühlborn LK, Rheinländer S, Weber L, Marzec B, Hellkamp M, Wieder P, Sax U, Kusch H, Nussbeck SY

7. API-based metadata extraction

A feature currently under development is REST API based metadata extraction. Antibody and publication metadata may be retrieved as JSON documents based on schema.org vocabulary definitions.

Feel free to try and test the following examples:

```
curl https://sfb1002.med.uni-goettingen.de/production/antibodies/api/2/antibody/
umg-sfb1002-antibody-secondary-649
```


```
▼ 0:
    version:                "2"
    @context:               "http://schema.org/"
    @type:                  "Thing"
    additionalType:         "Antibody"
    id:                     "649"
    pid:                    "umg-sfb1002-antibody-secondary-649"
  ▼ epic_pid:               "https://hdl.handle.net/11022/umg-sfb1002-antibody-secondary-649"
    research_group:         "AG Lehnart"
    lab_id:                 ""
    type:                   "secondary"
    antigen_symbol:         ""
    antibody_registry_id:   ""
    name:                   "Abberior STAR 580 goat anti-mouse"
    alternative_name:       ""
    tag_flurophore:         "Abberior STAR 580"
    raised_id:              "Goat"
    reacts_with:            ""
    clone:                  ""
    isotype:                "IgG"
    clonality:              ""
    demasking:              ""
    antigen:                "mouse antibodies"
    company:                "Abberior"
    catalog_no:             "2-0002-005-1"
    lot_no:                 ""
  ▼ description:            "Abberior STAR 580 is the dye of choice for orange fluorescence. Moreover, the dye is particularly
                            designed and tested for 2-color STED microscopy in combination with Abberior STAR 635 or STAR
                            635P.\r\nThe dye can most effectively be depleted in STED microscopy at 700 to 775 nm.\r\n"
    last_modified:          "Wagner, Dr., Eva"
```

```
curl -H "Accept: application/json"
"https://sfb1002.med.uni-goettingen.de/production/literature/publications/201"
```

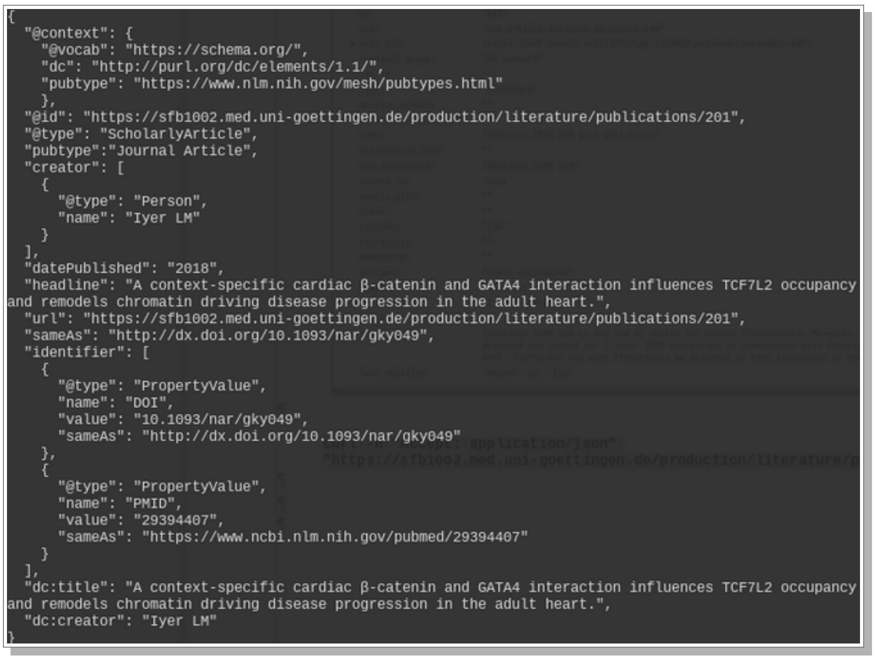

CC-BY-SA 4.0

Suhr M, Lehmann C, Bauer CR, Bender T, Knopp C, Freckmann LK, Öst Hansen B, Henke C, Aschenbrandt G, Kühlborn LK, Rheinländer S, Weber L, Marzec B, Hellkamp M, Wieder P, Sax U, Kusch H, Nussbeck SY
